# Comparison of usual care and the HEART score for effectively and safely discharging patients with low‐risk chest pain in the emergency department: would the score always help?

**DOI:** 10.1002/clc.23325

**Published:** 2019-12-23

**Authors:** Guangmei Wang, Wen Zheng, Shuo Wu, Jingjing Ma, He Zhang, Jiaqi Zheng, Jiali Wang, Feng Xu, Yuguo Chen

**Affiliations:** ^1^ Department of Emergency Medicine and Chest Pain Center Qilu Hospital of Shandong University Jinan China; ^2^ Clinical Research Center for Emergency and Critical Care Medicine of Shandong Province, Institute of Emergency and Critical Care Medicine of Shandong University Qilu Hospital of Shandong University Jinan China; ^3^ Key Laboratory of Emergency and Critical Care Medicine of Shandong Province, Key Laboratory of Cardiopulmonary‐Cerebral Resuscitation Research of Shandong Province Qilu Hospital of Shandong University Jinan China; ^4^ The Key Laboratory of Cardiovascular Remodeling and Function Research, Chinese Ministry of Education, Chinese Ministry of Health and Chinese Academy of Medical Sciences; The State and Shandong Province Joint Key Laboratory of Translational Cardiovascular Medicine Qilu Hospital of Shandong University Jinan China

**Keywords:** chest pain, discharge, emergency department, HEART, usual care

## Abstract

**Background:**

Triage decisions for chest pain patients receiving usual care are based on a dynamic and comprehensive strategy performed in the physician's mind. It remains controversial whether simple, structured risk tools can surpass real, complex judgments.

**Hypothesis:**

The potentially used History, Electrocardiogram, Age, Risk factors, Troponin (HEART) score would help identify low‐risk patients for discharge.

**Methods:**

Patients with acute, non‐traumatic chest pain managed according to usual care were consecutively enrolled in a tertiary university hospital in China from August 24, 2015 to September 30, 2017. Major adverse cardiac events (MACE) included death, acute myocardial infarction, revascularization, and significant coronary stenosis (>50%) within 30 days. We compared the efficacy and safety of usual care and the potentially used HEART score in this population.

**Results:**

Of 2185 patients analyzed, 926 (42.4%) patients were directly discharged by usual care, whereas HEART≤3 would have identified 524 (24.0%) patients as low‐risk (*P* < .001). The MACE rate in discharged patients was 2.2% (20/926) and would have been 5.2% (27/524) in those with HEART≤3 (*P* = .002). For discharged patients, the MACE rates in HEART≤3 vs HEART>3 groups were not significantly different (1.5% vs 2.7%, *P* = .225). Negative predictive value (NPV) was higher with usual care than with the HEART score (*P* = .003), but sensitivity was similar. For 340 patients with serial troponins, usual care was superior to the potentially used HEART score in regard to efficacy.

**Conclusions:**

At this institution, usual care identified many more patients for discharge than the HEART score would have without apparently different outcomes in discharged patients with lower vs higher HEART scores. The HEART score would not appear to provide helpful risk stratification.

## 1 INTRODUCTION

Acute chest pain is one of the most common reasons for emergency department (ED) evaluation. Only a small proportion of patients receive a final diagnosis of acute coronary syndrome (ACS).^1^, ^2^ Reliably detecting patients with ACS remains a diagnostic dilemma.^3^ Inappropriate admission of patients with benign disease is neither indicated nor cost‐effective, whereas inadvertent discharge of patients with ACS from the ED is associated with increased mortality and liability.^4^, ^5^ Previous studies have shown that between 2.1% and 4.6% of patients with acute myocardial infarction (AMI) in the ED are mistakenly discharged.^4^, ^6^, ^7^ Montassier et al found that 3.7% of patients were mistakenly discharged and presented with major adverse cardiac events (MACE) within 60 days.^8^ The accurate and safe identification of patients who can be directly discharged from the ED is a challenge for physicians.

Current guidelines recommend the use of structured risk stratification tools to evaluate and triage patients with suspected ACS presenting to the ED.^9^, ^10^, ^11^ Previous studies have indicated that the performance of the History, Electrocardiogram (ECG), Age, Risk factors, Troponin (HEART) score seems superior to other risk prediction scores.^12^, ^13^, ^14^, ^15^, ^16^ It has been recommended that patients with a HEART score ≤3 should be discharged without further diagnostic testing, including no second cardiac troponin (cTn) measurement.^17^, ^18^

However, triage decisions for chest pain patients using usual care are based on a comprehensive strategy performed in the physician's mind. After all, dynamic variations can appear in every aspect of a patient's clinical condition, including symptoms, signs, ECG, markers, intentions to receive care, and others. It takes time to observe these changes, but currently used risk stratification scores merely focus on one cross‐section of the timeline during an ED stay.^18^, ^19^, ^20^ To solve this problem, a triage pathway, such as the HEART pathway, based on a clinical score and two serial cTn tests have been developed and evaluated.^21^, ^22^ Only the change in cTn over time is considered. Therefore, it remains controversial whether simple, structured risk tools can surpass real, complex judgments.^15^, ^17^, ^23^

The performance of disposition decision protocols to discharge low‐risk patients mainly depends on efficacy (defined as the proportion of patients discharged) and safety (quantified by the sensitivity and negative predictive value [NPV] for MACE).^24^ In this study, we aimed to compare the efficacy and safety of usual care with the potentially used HEART score and pathway to identify patients for direct discharge in a tertiary hospital.

## 2 METHODS

### 2.1 Study population

This study was a retrospective analysis of a prospective observational cohort. Patients were consecutively recruited from the ED of Qilu Hospital of Shandong University, a tertiary university hospital that has 24 hours access to interventional angiography, between August 24, 2015 and September 30, 2017. All adult patients with acute, non‐traumatic chest pain or other symptoms suggestive of ACS occurring in the previous 24 hours and for whom the attending physician requested cTn for suspected ACS were included. Other symptoms suggestive of ACS may include shortness of breath, nausea, vomiting, jaw pain, and others.^25^ Patients were excluded if they were unwilling to provide informed consent, had an initial impression of ST‐segment elevation myocardial infarction (STEMI), died in the ED, were transferred to other hospitals or left against medical advice.

This study was approved by the ethics committee of Qilu Hospital of Shandong University, and all patients provided written informed consent.

### 2.2 Data collection

Clinical data were collected on a standardized case report form (CRF) in accordance with clinical data standards by trained research assistants.^25^ All the elements of the HEART score were included. The disposition after ED evaluation was categorized into discharged and undischarged. Undischarged included hospitalization and referral to a cardiologist. Hospitalization was defined as admission to an inpatient unit or to an observation room in the ED for at least 24 hours.^25^ Follow‐up after 30 days was conducted by trained research assistants to acquire information about adverse events, hospital revisits, and readmission over the telephone. The relevant medical records were obtained if a hospital admission was reported during the follow‐up period. Local death registry data were checked to ensure whether patients lost to contact were deceased.

### 2.3 Decision strategies

Every subject in this study was managed by usual care (discharged or undischarged) and retrospectively evaluated to be low or high risk according to the potentially used HEART score (≤3 or > 3).

Usual care was defined as the clinical practice of the physicians on duty. Direct discharge of a patient indicated stratifying the patient as low risk. Physicians assessed the risk of MACE in patients with suspected ACS by integrating patients' history; the results of a dynamic evaluation of symptoms, signs, ECG, and laboratory measurements; and their clinical expertise or intuition. Measurement of cTn for each patient was at the discretion of the attending physicians and not at established time intervals. No quantitative assessment approach was used. The length of stay (LOS) in the ED was calculated as the interval between discharge and presentation.

The methods for calculating the HEART score and HEART pathway have been previously described.^21^ The HEART score consisted of five elements: history, ECG, age, risk factors, and troponin. The history component was scored using a list of predefined chest pain characteristics that were categorized as typical or atypical. The ECG component was scored based on the impression of the first ECG by treating physicians. The first cTn results and the 99th percentile of the upper reference limit (URL) were used to calculate the scores. The overall HEART scores were retrospectively determined by the SAS program to guarantee their veracity and consistency. Patients with a HEART score of 0 to 3 were categorized as low‐risk of developing MACE and considered eligible for direct discharge from the ED without further diagnostic testing. The HEART pathway (−) indicated patients were low risk if their HEART score was ≤3 and two cTn tests were both negative (the first and second cTn values after presentation). Since the use of the HEART score would have indicated discharge immediately after the low‐risk score was assigned, the time of the initial cTn report (completing the HEART score) was taken as the discharge time in the HEART score group.

### 2.4 Clinical outcome

The primary outcome was MACE within 30 days after initial presentation, including death from all causes, index (being the cause for the initial presentation) or subsequent (occurring during the follow‐up) AMI, revascularization (emergency/urgent/elective percutaneous coronary intervention [PCI] or coronary artery bypass grafting [CABG]), and coronary angiography revealing significant stenosis (>50%) with conservative treatment. The secondary outcome was the composite of death from all causes, index or subsequent AMI and emergency revascularization within 30 days. Each event in MACE was adjudicated by two independent cardiologists for all patients in accordance with the definitions following a review of all available medical records.^25^, ^26^, ^27^, ^28^ In case of disagreement, a third cardiologist reviewed the record. AMI referred to a type 1 myocardial infarction and was defined as myocardial necrosis in the context of myocardial ischemia due to a definite or highly suspected plaque rupture and coronary thrombosis. Myocardial necrosis was diagnosed based on the rise and/or fall (a delta of ≥20% was used) of cTn with at least one value above the diagnostic threshold.^10^, ^28^

### 2.5 Statistical analysis

Continuous variables are presented as the mean ± SDs or median (interquartile range), and categorical variables are summarized as numbers and percentages. The outcome rates were compared with a *χ* ^2^ test between groups. Diagnostic accuracy with a 95% confidence interval (CI) for usual care and the potentially used HEART score or pathway for MACE were determined, including sensitivity, specificity, NPV, and positive predictive value (PPV). The efficacy (proportion of patients identified as low risk), sensitivity, and specificity of the different strategies were compared using the McNemar test based on paired 4‐fold tables.^29^ The NPV and PPV of the different strategies were compared using χ^2^ tests for the respective proportions. A *P*‐value less than .05 (two‐sided significance testing) was considered statistically significant. All statistical analyses were performed using SAS V.9.4 (SAS Institute, Inc., Cary, North Carolina).

## 3 RESULTS

### 3.1 Study population

**Figure 1:**
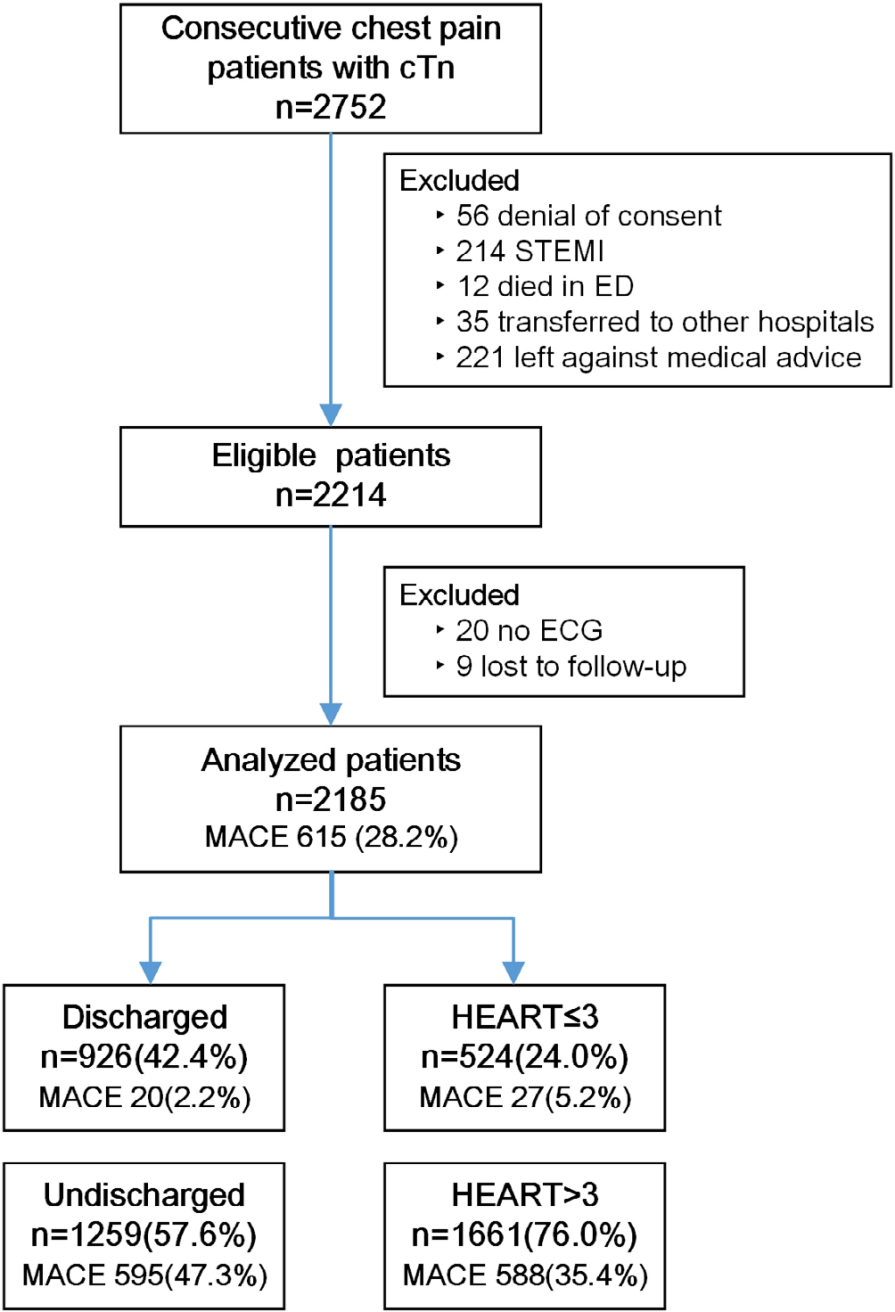
Flowchart used for patient analysis. cTn, cardiac troponin; ECG, electrocardiogram; ED, emergency department; HEART, History, ECG, Age, Risk factors, Troponin; MACE, major adverse cardiac events; STEMI, ST‐segment elevation myocardial infarction

A total of 2752 patients with acute, non‐traumatic chest pain, and initial cTn tests presented to the ED of Qilu Hospital from August 24, 2015 to September 30, 2017. Patients were excluded for denial of informed consent (56), an initial diagnosis of STEMI (214), dying in the ED (12), being transferred to other hospitals (35), or leaving against medical advice (221). There were 20 patients with insufficient information to calculate the HEART score, and nine patients were lost to follow‐up. The remaining 2185 patients were included in the analysis (Figure 1). The baseline characteristics of the entire analyzed cohort are presented in Table 1.

**Table 1:** Baseline characteristics of the analyzed cohort

	Total n = 2185
Age (y), mean ± SD	63.8 ± 13.6
Male, n (%)	1096 (50.2)
Risk factors, n (%)	
Current smoker	313 (14.3)
Obesity (BMI ≥ 28 kg/m^2^)	420 (19.2)
Diabetes	560 (25.6)
Hypertension	1320 (60.4)
Hyperlipidemia	222 (10.2)
Family history of premature CAD	391 (17.9)
Medical history, n (%)	
MI	500 (22.9)
Catheterization with stenosis ≥50%	584 (26.7)
PCI	449 (20.5)
CABG	53 (2.4)
PAD	3 (0.1)
Stroke	295 (13.5)
Vital signs at presentation, mean ± SD	
SBP (mm Hg)	150.5 ± 26.5
DBP (mm Hg)	83.8 ± 16.0
HR (bpm)	80.4 ± 18.7
Negative troponin, n (%)	1780 (81.5)
Normal ECG, n (%)	669 (30.6)
LOS (h), median (IQR)	9.4 (4.0,23.0)
HEART, median (IQR)	5 (4,6)
HEART ≤ 3, n (%)	524 (24.0)
Discharged, n (%)	926 (42.4)

Abbreviations: BMI, body mass index; CABG, coronary artery bypass grafting; CAD, coronary artery disease; DBP, diastolic blood pressure; ECG, electrocardiography; HEART, History, ECG, Age, Risk factors, Troponin; HR, heart beat; IQR, interquartile range; LOS, length of stay; MI, myocardial infarction; PAD, peripheral arterial disease; PCI, percutaneous coronary intervention; SBP, systolic blood pressure.

### 3.2 Efficacy of usual care vs the HEART score

Usual care triaged 926 (42.4%) patients to be directly discharged without further testing. If the HEART score was used in the entire cohort, 524 (24.0%) patients would have been identified as low risk (Figure 1). Based on the paired 4‐fold table stratifying chest pain by usual care vs the potentially used HEART score (Table S1), the difference between these two percentages was significant (*P* < .001). The specificity of usual care to rule in events was 0.577 (0.553, 0.602), which was superior to the HEART score with 0.317 (0.294, 0.340) (*P* < .001) (Table 2). The baseline characteristics of the low‐ vs high‐risk groups categorized by usual care or the potentially used HEART score are shown in Table S2, and the median LOS of patients discharged by usual care was 5.5 (1.7, 8.7) hours, which was longer than the assumed time using a HEART score ≤ 3, 1.5 hours (1.4,1.7). For the composite of death, AMI and emergency revascularization, the specificity of usual care outperformed the HEART score (Table S3).

**Table 2:** Diagnostic accuracy for 30‐day MACE of usual care and the potentially used HEART score

	Usual care	HEART	*P*‐value
Sensitivity	0.967 (0.953,0.981)	0.956 (0.940,0.972)	.311
NPV	0.978 (0.969,0.988)	0.948 (0.930,0.967)	.003
Specificity	0.577 (0.553,0.602)	0.317 (0.294,0.340)	<.001
PPV	0.473 (0.445,0.500)	0.354 (0.331,0.377)	<.001

Abbreviations: HEART, History, ECG, Age, Risk factors, Troponin; NPV, negative predictive value; PPV, positive predictive value.

### 3.3 Outcomes

A total of 615 (28.2%) patients had 30‐day MACE in this chest pain cohort (Figure 1). As shown in Table 3, the MACE rate in patients discharged by usual care was 2.2% (20/926), and the rate would have been 5.2% (27/524) in those with a low HEART score (*P* = .002). For patients deemed to be low risk by usual care (discharged), the MACE rates in the HEART score ≤ 3 vs HEART score > 3 groups were not significantly different (1.5% vs 2.7%, *P* = .225). For patients with a low HEART score, the MACE rate in the discharged group was much lower than that in the undischarged group (1.5% vs 17.1%, *P* < .001). The incidence of the composite of death, AMI, and emergency revascularization was similar in patients discharged by usual care and in patients with the potentially used HEART score ≤ 3 (0.5% vs 1.5%, *P* = .079) (Table 3).

**Table 3:** Outcomes in low‐risk patients identified by usual care (discharged) and the potentially used HEART (≤3)

	Discharged				HEART ≤3				
	Total n = 926	HEART≤3 n = 401	HEART>3 n = 525	*P‐*value	Total n = 524	Discharged n = 401	Undischarged n = 123	*P‐*value	*P*‐value *
MACE, n (%)	20 (2.2)	6 (1.5)	14 (2.7)	.225	27 (5.2)	6 (1.5)	21 (17.1)	<.001	.002
Index AMI	3 (0.3)	1 (0.2)	2 (0.4)	1.000	7 (1.3)	1 (0.2)	6 (4.9)	.001	.042
Subsequent AMI	2 (0.2)	0 (0)	2 (0.4)	.508	1 (0.2)	0 (0)	1 (0.8)	.235	1.000
Death	0 (0)	0 (0)	0 (0)	—	0 (0)	0 (0)	0 (0)	—	—
Emergency PCI	1 (0.1)	0 (0)	1 (0.2)	1.000	1 (0.2)	0 (0)	1 (0.8)	.235	1.000
Urgent/elective PCI	14 (1.5)	4 (1)	10 (1.9)	.262	15 (2.9)	4 (1)	11 (8.9)	<.001	.078
CABG	0 (0)	0 (0)	0 (0)	—	0 (0)	0 (0)	0 (0)	—	—
Conservatively treated stenosis (>50%)	3 (0.3)	1 (0.2)	2 (0.4)	1.000	7 (1.3)	1 (0.2)	6 (4.9)	<.001	.042
Composite of death, AMI and emergency revascularization	5 (0.5)	1 (0.2)	4 (0.8)	.396	8 (1.5)	1 (0.2)	7 (5.7)	<.001	.079

Abbreviations: AMI, acute myocardial infarction; CABG, coronary artery bypass grafting; HEART, History, ECG, Age, Risk factors, Troponin; MACE, major adverse cardiac events; PCI, percutaneous coronary intervention.

* *P*‐value for usual care (discharged) vs the potentially used HEART (≤3).

### 3.4 Safety of usual care vs the HEART score

The sensitivity of usual care was 0.967 (0.953, 0.981) and would have been 0.956 (0.940, 0.972) for the potentially used HEART score (*P =* .311). The difference in NPV was significant (*P* = .003) between usual care at 0.978 (0.969, 0.988) and the HEART score at 0.948 (0.930, 0.967) (Table 2). For the composite of death, AMI, and emergency revascularization, the sensitivity and NPV showed no significant differences between the two strategies (Table S3).

### 3.5 Performance of usual care vs the HEART pathway

As shown in Table 4, among 340 patients who received serial cTn tests, the proportion of low‐risk chest pain identified by usual care was 25.9% (88/340), whereas use of the HEART pathway would have noted only 11.5% (39/340) of patients as low risk (*P* < .001). The MACE rate in discharged patients was similar to that would have been in the HEART pathway low‐risk group (3.4% vs 7.7%, *P* = .370) (Table 4). For discharged patients, the difference in the MACE rates between the HEART pathway (−) and HEART pathway (+) groups was not significant (0% vs 4.7%, *P* = .559). For patients with a negative HEART pathway, the MACE rate in the discharged group was lower than that in the undischarged group (0% vs 20.0%, *P* < .050) (Table S4). The corresponding specificities of these two strategies were 0.445 (0.375, 0.516) and 0.188 (0.133, 0.244) (*P* < .001). The sensitivities were equal at 0.980 (0.957, 1.000), and NPV showed no difference at 0.966 (0.928, 1.000) and 0.923 (0.839, 1.000), respectively (*P* = .370) (Table 4).

**Table 4:** Performance of usual care and the potentially used HEART pathway in patients with serial troponin tests

	Usual care	HEART pathway	*P*‐value
Low‐risk patients/total patients (%)	88/340 (25.9)	39/340 (11.5)	<.001
MACE rate in low‐risk patients, n (%)	3 (3.4)	3 (7.7)	.370
Sensitivity	0.980 (0.957,1.000)	0.980 (0.957,1.000)	1.000
NPV	0.966 (0.928,1.000)	0.923 (0.839,1.000)	.370
Specificity	0.445 (0.375,0.516)	0.188 (0.133,0.244)	<.001
PPV	0.579 (0.518,0.640)	0.485 (0.429,0.542)	.032

Abbreviations: AMI, acute myocardial infarction; CABG, coronary artery bypass grafting; HEART, History, ECG, Age, Risk factors, Troponin; MACE, major adverse cardiac events; NPV, negative predictive value; PCI, percutaneous coronary intervention; PPV, positive predictive value.

## 4 DISCUSSION

This study was a retrospective analysis of a prospective, consecutive cohort of patients with acute, non‐traumatic chest pain, or other symptoms suggestive of ACS presenting to the ED. The efficacy and safety of usual care and the potentially used HEART strategies to discharge low‐risk patients were compared. In this Chinese tertiary hospital, usual care identified a much larger proportion of patients to directly discharge needing no further investigations than the HEART score would have. The rate of MACE in discharged patients was lower than that would have been in those with a low HEART score (≤3). For discharged patients, the high HEART score (>3) group showed no apparent difference in outcomes compared with the low HEART score (≤3) group. On the contrary, for patients with a low HEART score, those deemed low risk by usual care (discharged) did have lower MACE rate than high‐risk (undischarged) patients.

Previous studies have demonstrated that clinician gestalt alone is insufficient or not superior for identifying patients who are safe to discharge from the ED.^15^, ^23^, ^30^, ^31^ However, the unstructured assessment used in these studies is generally a scale or an impression code of ACS probability completed based on history, symptoms, and physical examination performed by physicians. It has been shown that medical history, risk factors, symptoms, and physical examination do not have a sufficient discriminatory ability to “rule‐in” or “rule‐out” ACS in the ED.^32^ Furthermore, this kind of gestalt is mainly presented as an initial judgment after patients' arrival and does not convey the sophisticated evaluation performed after making a series of observations of various aspects before the discharge decision. Although usual care in this study cannot be structurally described, it combined clinical manifestations with the initial ECG and cTn levels and, if necessary, with the serial ECG and cTn levels. Importantly, physicians following usual care should pay close attention to any changes that occur in emotions, symptoms, signs, ECG, markers, or other conditions.

If low‐risk patients are identified and directly discharged without compromising safety, the family, and health care burdens are significantly reduced.^4^, ^33^ Therefore, assessment approaches to stratify patients with acute chest pain have been heavily researched.^32^ Like other risk stratification models, HEART is a structured objective scoring tool to help physicians confidently select dischargeable patients without further diagnostic testing.^18^ This score consists of five elements, history, ECG, age, risk factors, and troponin, each of which can be assigned 0, 1, or 2 points. A previous randomized trial observed non‐inferior safety and no significant difference in early discharge when using the HEART score compared with usual care. The limited effect on health care resources was possibly due to physicians' hesitation to adhere to management recommendations from HEART.^17^ To some extent, this hesitation demonstrated a possible conflict between physicians' subjective judgment and the objective scores. In our cohort, the efficacy of usual care was superior to the potentially used HEART score with a lower MACE incidence. Patients who were discharged were older, with more risk factors and atherosclerosis than patients who would have been categorized as low‐risk by the HEART score (≤3). Again, this finding showed that history did not help to stratify the risk level of undifferentiated chest pain. Additionally, the HEART score is a snapshot of the patient's initial condition rather than a complete picture of the ED stay. Undoubtedly, a snapshot is much faster and cheaper, but not all patients provide a complete picture at presentation. A higher rate of missed index AMI and conservatively treated stenosis demonstrated that the snapshot lacked the ability to identify important information hidden in changes. A higher HEART score (>3) would not have indicated a higher MACE rate than a lower HEART score (≤3) in discharged patients. Although usual care was more time‐consuming to perform than determining the HEART score, for the low‐risk group, making a decision sooner may be not better.

Since cTn serves as the cornerstone of diagnosing AMI and has a kinetic release profile, serial cTn tests have been incorporated into clinical scores to make the assessment models safer, such as the HEART pathway.^21^ In our study, when serial cTn tests were considered, usual care was still superior to the potentially used HEART pathway in regard to efficacy. It is understandable that ignorance of the dynamic changes in complex aspects other than cTn may indicate an area to improve for risk scores and pathways.

It should be mentioned that to capture all clinically relevant end points, urgent/elective PCI, CABG, and coronary angiography revealing significant stenosis were included in our definition of MACE. Varying definitions of MACE could lead to different results.^34^ If MACE only included all‐cause death, AMI, and emergency revascularization, the incidence of MACE in patients discharged by usual care would have decreased from 2.2% to 0.5% (5/926), with a sensitivity of up to 98.9% and NPV of 99.5%. This level of safety would be acceptable (>99%) to most physicians according to an international survey.^35^ The specificity of usual care still outperformed the HEART score with a similar level of safety. However, safe discharge is not only the avoidance of acute critical events (like AMI or death) but also potential events to which coronary stenosis may lead. Therefore, it is more appropriate to cover all these events to evaluate the performance of strategies to directly discharge chest pain patients without further diagnostic testing.^17^ A meta‐analysis demonstrated that a HEART score of 0 to 3 could miss 1.6% MACE (range 0.9%‐5.9%) with a pooled sensitivity and specificity of 96.7% (range 75.5%‐100%) and 47.0% (range 31.8%‐67.5%), respectively.^36^ In our analysis, the incidence of MACE in patients with a low HEART score would have been much higher (5.2%) with lower sensitivity and specificity than the pooled values mentioned above. The possible reasons for these disparities may be the different definition of MACE and ACS prevalence.^32^, ^37^ After all, the percent of patients suffering 30‐day MACE in our study was 28.2%, which was higher than that in most prior research.^36^

There were several limitations in the study. First, this study was a single‐center observational study, which may limit the generalizability of the findings. The performance of usual care and clinical scores in broader patient populations should be determined by further studies of heterogeneous groups. In particular, the findings need to be confirmed in a randomized trial. Our report may provide implications for improving clinical risk stratification tools, of which objectivity and repeatability may overcome the variability between physicians and between hospitals. Second, we did not invite the discharged patients to take cTn tests and ECGs in a follow‐up assessment. Therefore, it was possible that the incidence of MACE was underestimated. However, to reduce this underestimation, we reviewed all relevant medical records to determine the occurrence of any outcome if patients revisited a hospital during the follow‐up period.

## 5 CONCLUSION

At this institution, usual care identified a much larger proportion of patients to directly discharge needing no further investigations than the potentially used HEART strategies without compromising safety. There was no apparent difference in outcomes in the lower vs higher HEART score groups for the patients discharged by usual care. Compared to a physician's dynamic and comprehensive assessment of each patient's individual information, application of the HEART score would not appear to provide helpful risk stratification.

## CONFLICT OF INTERESTS

The authors declare no potential conflict of interests.

## AUTHOR CONTRIBUTIONS

Yuguo Chen, Jiali Wang, and Feng Xu contributed to the initiation, planning, and conduction of the study. Wen Zheng, Guangmei Wang, Jingjing Ma, He Zhang, and Jiaqi Zheng conducted the study supervision. Guangmei Wang, Wen Zheng and Shuo Wu did the analysis and interpretation of data. Guangmei Wang and Wen Zheng drafted the manuscript. Guangmei Wang and Wen Zheng contributed equally to this work.

## Supporting information


**Table S1** Agreement of the potentially used HEART score with usual care for stratifying chest pain.

**Table S2** Baseline characteristics of patients stratified by usual care and the potentially used HEART score.

**Table S3** Diagnostic accuracy for the composite of death, AMI and emergency revascularization of usual care and the potentially used HEART score.

**Table S4** Outcomes in low‐risk patients with serial troponin tests identified by usual care (discharged) and the potentially used HEART pathway (−).

## References

[1] Niska R , Bhuiya F , Xu J , National Hospital Ambulatory Medical Care Survey . Emergency department summary. Natl Health Stat Report. 2007;2010:1‐31. 20726217

[2] Lindsell CJ , Anantharaman V , Diercks D , et al. The internet tracking registry of acute coronary syndromes (i*trACS): a multicenter registry of patients with suspicion of acute coronary syndromes reported using the standardized reporting guidelines for emergency department chest pain studies. Ann Emerg Med. 2006;48:666‐677. 17014928 10.1016/j.annemergmed.2006.08.005

[3] Pierce MA , Hess EP , Kline JA , et al. The chest pain choice trial: a pilot randomized trial of a decision aid for patients with chest pain in the emergency department. Trials. 2010;11:57. 20478056 10.1186/1745-6215-11-57 PMC2881067

[4] Pope JH , Aufderheide TP , Ruthazer R , et al. Missed diagnoses of acute cardiac ischemia in the emergency department. N Engl J Med. 2000;342:1163‐1170. 10770981 10.1056/NEJM200004203421603

[5] Weinstock MB , Weingart S , Orth F , et al. Risk for clinically relevant adverse cardiac events in patients with chest pain at hospital admission. JAMA Intern Med. 2015;175:1207‐1212. 25985100 10.1001/jamainternmed.2015.1674

[6] Schull MJ , Vermeulen MJ , Stukel TA . The risk of missed diagnosis of acute myocardial infarction associated with emergency department volume. Ann Emerg Med. 2006;48:647‐655. 17112926 10.1016/j.annemergmed.2006.03.025

[7] Christenson J , Innes G , McKnight D , et al. Safety and efficiency of emergency department assessment of chest discomfort. CMAJ. 2004;170:1803‐1807. 15184334 10.1503/cmaj.1031315 PMC419767

[8] Montassier E , Batard E , Gueffet JP , Trewick D , le Conte P . Outcome of chest pain patients discharged from a French emergency department: a 60‐day prospective study. J Emerg Med. 2012;42:341‐344. 21247726 10.1016/j.jemermed.2010.11.036

[9] Roffi M , Patrono C , Collet JP , et al. 2015 ESC guidelines for the management of acute coronary syndromes in patients presenting without persistent ST‐segment elevation: task force for the Management of Acute Coronary Syndromes in patients presenting without persistent ST‐segment elevation of the European Society of Cardiology (ESC). Eur Heart J. 2016;37:267‐315. 26320110 10.1093/eurheartj/ehv320

[10] Amsterdam EA , Wenger NK , Brindis RG , et al. 2014 AHA/ACC guideline for the management of patients with non‐ST‐elevation acute coronary syndromes: a report of the American College of Cardiology/American Heart Association task force on practice guidelines. Circulation. 2014;130:e344‐e426. 25249585 10.1161/CIR.0000000000000134

[11] Chew DP , Scott IA , Cullen L , et al. National Heart Foundation of Australia & Cardiac Society of Australia and New Zealand: Australian clinical guidelines for the Management of Acute Coronary Syndromes 2016. Heart Lung Circ. 2016;25:895‐951. 27476580 10.1016/j.hlc.2016.06.789

[12] Backus BE , Six AJ , Kelder JC , et al. A prospective validation of the HEART score for chest pain patients at the emergency department. Int J Cardiol. 2013;168:2153‐2158. 23465250 10.1016/j.ijcard.2013.01.255

[13] Sakamoto JT , Liu N , Koh ZX , et al. Comparing HEART, TIMI, and GRACE scores for prediction of 30‐day major adverse cardiac events in high acuity chest pain patients in the emergency department. Int J Cardiol. 2016;221:759‐764. 27428317 10.1016/j.ijcard.2016.07.147

[14] Poldervaart JM , Langedijk M , Backus BE , et al. Comparison of the GRACE, HEART and TIMI score to predict major adverse cardiac events in chest pain patients at the emergency department. Int J Cardiol. 2017;227:656‐661. 27810290 10.1016/j.ijcard.2016.10.080

[15] Mahler SA , Miller CD , Hollander JE , et al. Identifying patients for early discharge: performance of decision rules among patients with acute chest pain. Int J Cardiol. 2013;168:795‐802. 23117012 10.1016/j.ijcard.2012.10.010 PMC3565031

[16] Carlton E , Body R , Greaves K . External validation of the Manchester acute coronary syndromes decision rule. Acad Emerg Med. 2016;23:136‐143. 26802433 10.1111/acem.12860

[17] Poldervaart JM , Reitsma JB , Backus BE , et al. Effect of using the HEART score in patients with chest pain in the emergency department: a stepped‐wedge. Cluster Randomized Trial Ann Intern Med. 2017;166:689‐697. 28437795 10.7326/M16-1600

[18] Six AJ , Backus BE , Kelder JC . Chest pain in the emergency room: value of the HEART score. Neth Heart J. 2008;16:191‐196. 18665203 10.1007/BF03086144 PMC2442661

[19] Antman EM , Cohen M , Bernink PJ , et al. The TIMI risk score for unstable angina/non‐ST elevation MI: a method for prognostication and therapeutic decision making. JAMA. 2000;284:835‐842. 10938172 10.1001/jama.284.7.835

[20] Granger CB , Goldberg RJ , Dabbous O , et al. Predictors of hospital mortality in the global registry of acute coronary events. Arch Intern Med. 2003;163:2345‐2353. 14581255 10.1001/archinte.163.19.2345

[21] Mahler SA , Riley RF , Hiestand BC , et al. The HEART pathway randomized trial: identifying emergency department patients with acute chest pain for early discharge. Circ Cardiovasc Qual Outcomes. 2015;8:195‐203. 25737484 10.1161/CIRCOUTCOMES.114.001384 PMC4413911

[22] Than M , Aldous S , Lord SJ , et al. A 2‐hour diagnostic protocol for possible cardiac chest pain in the emergency department: a randomized clinical trial. JAMA Intern Med. 2014;174:51‐58. 24100783 10.1001/jamainternmed.2013.11362

[23] Visser A , Wolthuis A , Breedveld R , ter Avest E . HEART score and clinical gestalt have similar diagnostic accuracy for diagnosing ACS in an unselected population of patients with chest pain presenting in the ED. Emerg Med J. 2015;32:595‐600. 25217099 10.1136/emermed-2014-203798

[24] Morawiec B , Boeddinghaus J , Wussler D , et al. Modified HEART score and high‐sensitivity cardiac troponin in patients with suspected acute myocardial infarction. J Am Coll Cardiol. 2019;73:873‐875. 30784680 10.1016/j.jacc.2018.12.013

[25] Cannon CP , Brindis RG , Chaitman BR , et al. 2013 ACCF/AHA key data elements and definitions for measuring the clinical management and outcomes of patients with acute coronary syndromes and coronary artery disease: a report of the American College of Cardiology Foundation/American Heart Association task force on clinical data standards (Writing Committee to Develop Acute Coronary Syndromes and Coronary Artery Disease Clinical Data Standards). Circulation. 2013;127:1052‐1089. 23357718 10.1161/CIR.0b013e3182831a11

[26] Cullen L , Than M , Brown AF , et al. Comprehensive standardized data definitions for acute coronary syndrome research in emergency departments in Australasia. Emerg Med Australas. 2010;22:35‐55. 20136639 10.1111/j.1742-6723.2010.01256.x

[27] Hicks KA , Tcheng JE , Bozkurt B , et al. 2014 ACC/AHA key data elements and definitions for cardiovascular endpoint events in clinical trials: a report of the American College of Cardiology/American Heart Association task force on clinical data standards (Writing Committee to Develop Cardiovascular Endpoints Data Standards). J Am Coll Cardiol. 2015;66:403‐469. 25553722 10.1016/j.jacc.2014.12.018

[28] Thygesen K , Alpert JS , Jaffe AS , et al. Third universal definition of myocardial infarction. Eur Heart J. 2012;33:2551‐2567. 22922414 10.1093/eurheartj/ehs184

[29] Fagerland MW , Lydersen S , Laake P . The McNemar test for binary matched‐pairs data: mid‐p and asymptotic are better than exact conditional. BMC Med Res Methodol. 2013;13:91. 23848987 10.1186/1471-2288-13-91 PMC3716987

[30] Chandra A , Lindsell CJ , Limkakeng A , et al. Emergency physician high pretest probability for acute coronary syndrome correlates with adverse cardiovascular outcomes. Acad Emerg Med. 2009;16:740‐748. 19673712 10.1111/j.1553-2712.2009.00470.x

[31] Body R , Cook G , Burrows G , Carley S , Lewis PS . Can emergency physicians ‘rule in’ and ‘rule out’ acute myocardial infarction with clinical judgement? Emerg Med J. 2014;31:872‐876. 25016388 10.1136/emermed-2014-203832

[32] Hollander JE , Than M , Mueller C . State‐of‐the‐art evaluation of emergency department patients presenting with potential acute coronary syndromes. Circulation. 2016;134:547‐564. 27528647 10.1161/CIRCULATIONAHA.116.021886

[33] Six AJ , Backus BE , Kingma A , Kaandorp SI . Consumption of diagnostic procedures and other cardiology care in chest pain patients after presentation at the emergency department. Neth Heart J. 2012;20:499‐504. 23090421 10.1007/s12471-012-0322-6 PMC3515734

[34] Kip KE , Hollabaugh K , Marroquin OC , Williams DO . The problem with composite end points in cardiovascular studies: the story of major adverse cardiac events and percutaneous coronary intervention. J Am Coll Cardiol. 2008;51:701‐707. 18279733 10.1016/j.jacc.2007.10.034

[35] Than M , Herbert M , Flaws D , et al. What is an acceptable risk of major adverse cardiac event in chest pain patients soon after discharge from the emergency department?: a clinical survey. Int J Cardiol. 2013;166:752‐754. 23084108 10.1016/j.ijcard.2012.09.171

[36] Van Den Berg P , Body R . The HEART score for early rule out of acute coronary syndromes in the emergency department: a systematic review and meta‐analysis. Eur Heart J Acute Cardiovasc Care. 2018;7:111‐119. 28534694 10.1177/2048872617710788

[37] Wang G , Wang J , Wu S , et al. Clinical impact of using a more sensitive troponin assay in patients with acute chest pain. Clin Cardiol. 2019;42:561‐567. 30887538 10.1002/clc.23177 PMC6522991

