# Supplementary material for: Comparison of usual care and the HEART score for effectively and safely discharging patients with low‐risk chest pain in the emergency department: would the score always help?
Source: Clin Cardiol. 2019 Dec 23;43(4):371–8. doi: 10.1002/clc.23325 (PMC7144490; doi:10.1002/clc.23325)
Supplement: Supplementary file 1 — Table S1 Agreement of the potentially used HEART score with usual care for stratifying chest pain. [file CLC-43-371-s001.docx]

**Supplementary Table 1.** Agreement of the potentially used HEART score with usual care for stratifying chest pain.

|  |  | **Usual care** | | **Total** |
| --- | --- | --- | --- | --- |
|  |  | **Low-risk (Discharged)** | **High-risk**  **(Undischarged)** |  |
| **HEART score** | **Low-risk (≤3)** | 401 | 123 | 524 |
|  | **High-risk (>3)** | 525 | 1136 | 1661 |
| **Total** | | 926 | 1259 | 2185 |

HEART, History, ECG, Age, Risk factors, Troponin.
