# Supplementary material for: Comparison of usual care and the HEART score for effectively and safely discharging patients with low‐risk chest pain in the emergency department: would the score always help?
Source: Clin Cardiol. 2019 Dec 23;43(4):371–8. doi: 10.1002/clc.23325 (PMC7144490; doi:10.1002/clc.23325)
Supplement: Supplementary file 2 — Table S2 Baseline characteristics of patients stratified by usual care and the potentially used HEART score. [file CLC-43-371-s002.docx]

**Supplementary Table 2**. Baseline characteristics of patients stratified by usual care and the potentially used HEART score.

|  | **Usual care** | |  | **HEART** | |
| --- | --- | --- | --- | --- | --- |
|  | **Discharged**  **n=926** | **No discharged**  **n=1259** |  | **HEART≤3**  **n=524** | **HEART>3**  **n=1661** |
| **Age (y), mean ± SD** | 60.1±14.5 | 66.5±12.1 |  | 52.2±13.7 | 67.5±11.3 |
| **Male, n (%)** | 430(46.4) | 666(52.9) |  | 275(52.5) | 821(49.4) |
| **Risk factors, n (%)** |  |  |  |  |  |
| Current smoker | 120(13) | 193(15.3) |  | 89(17) | 224(13.5) |
| Obesity (BMI≥28 kg/m2) | 170(18.4) | 250(19.9) |  | 86(16.4) | 334(20.1) |
| Diabetes | 183(19.8) | 377(29.9) |  | 38(7.3) | 522(31.4) |
| Hypertension | 474(51.2) | 846(67.2) |  | 159(30.3) | 1161(69.9) |
| Hyperlipidemia | 100(10.8) | 122(9.7) |  | 33(6.3) | 189(11.4) |
| Family history of premature CAD | 170(18.4) | 221(17.6) |  | 68(13) | 323(19.4) |
| **Medical history, n (%)** |  |  |  |  |  |
| MI | 140(15.1) | 360(28.6) |  | 14(2.7) | 486(29.3) |
| Catheterization with stenosis ≥50% | 192(20.7) | 392(31.1) |  | 19(3.6) | 565(34) |
| PCI | 154(16.6) | 295(23.4) |  | 14(2.7) | 435(26.2) |
| CABG | 10(1.1) | 43(3.4) |  | 0(0) | 53(3.2) |
| PAD | 0(0) | 3(0.2) |  | 0(0) | 3(0.2) |
| Stroke | 84(9.1) | 211(16.8) |  | 7(1.3) | 288(17.3) |
| **Vital signs at presentation, mean ± SD** |  |  |  |  |  |
| SBP (mmHg) | 150.0±25.3 | 150.8±27.3 |  | 146.0±23.6 | 151.9±27.2 |
| DBP (mmHg) | 83.7±14.6 | 83.9±17.0 |  | 85.4±15.0 | 83.3±16.3 |
| HR (bpm) | 79.5±16.4 | 81.1±20.2 |  | 79.4±14.7 | 80.7±19.8 |
| **Negative Troponin, n (%)** | 909(98.2) | 871(69.2) |  | 517(98.7) | 1263(76) |
| **Normal ECG, n (%)** | 464(50.1) | 205(16.3) |  | 409(78.1) | 260(15.7) |
| **LOS (h), median (IQR)** | 5.5(1.7,8.7) | 18.3(8.2,39.3) |  | 1.5(1.4,1.7)* | 1.5(1.3,1.7)* |
| **HEART, median (IQR)** | 4(3,5) | 6(5,7) |  | 2(2,3) | 6(5,7) |
| **HEART≤3, n (%)** | 401(43.3) | 123(9.8) |  | - | - |
| **Discharged, n (%)** | - | - |  | 401(76.5) | 525(31.6) |

BMI, body mass index; CABG, coronary artery bypass grafting; CAD, coronary artery disease; DBP, diastolic blood pressure; ECG, electrocardiography; HEART, History, ECG, Age, Risk factors, Troponin; HR, heart beat; IQR, interquartile range; LOS, length of stay; MI, myocardial infarction; PAD, peripheral arterial disease; PCI, percutaneous coronary intervention; SBP, systolic blood pressure; SD, standard deviation.

* The time of the initial troponin report was taken as the discharge time in this group
