# Supplementary material for: Comparison of usual care and the HEART score for effectively and safely discharging patients with low‐risk chest pain in the emergency department: would the score always help?
Source: Clin Cardiol. 2019 Dec 23;43(4):371–8. doi: 10.1002/clc.23325 (PMC7144490; doi:10.1002/clc.23325)
Supplement: Supplementary file 3 — Table S3 Diagnostic accuracy for the composite of death, AMI and emergency revascularization of usual care and the potentially used HEART score. [file CLC-43-371-s003.docx]

**Supplementary Table 3.** Diagnostic accuracy for the composite of death, AMI and emergency revascularization of usual care and the potentially used HEART score.

|  | **Usual care** | **HEART** | ***P* value** |
| --- | --- | --- | --- |
| **Sensitivity** | 0.989(0.979,0.999) | 0.982(0.969,0.994) | 0.549 |
| **NPV** | 0.995(0.990,0.999) | 0.985(0.974,0.995) | 0.079 |
| **Specificity** | 0.527(0.504,0.551) | 0.295(0.274,0.317) | <0.001 |
| **PPV** | 0.344(0.318,0.370) | 0.259(0.238,0.280) | <0.001 |

AMI, acute myocardial infarction; HEART, History, ECG, Age, Risk factors, Troponin; NPV, negative predictive value; PPV, positive predictive value.
