# Supplementary material for: Comparison of usual care and the HEART score for effectively and safely discharging patients with low‐risk chest pain in the emergency department: would the score always help?
Source: Clin Cardiol. 2019 Dec 23;43(4):371–8. doi: 10.1002/clc.23325 (PMC7144490; doi:10.1002/clc.23325)
Supplement: Supplementary file 4 — Table S4 Outcomes in low‐risk patients with serial troponin tests identified by usual care (discharged) and the potentially used HEART pathway (−). [file CLC-43-371-s004.docx]

**Supplementary Table 4.** Outcomes in low-risk patients with serial troponin tests identified by usual care (discharged) and the potentially used HEART pathway (-).

|  | **Discharged** | | | |  | **HEART pathway (-) *** | | | |  | ***P* value **** |
| --- | --- | --- | --- | --- | --- | --- | --- | --- | --- | --- | --- |
|  | **Total**  **n=88** | **HEART pathway(-)**  **n=24** | **HEART pathway(+)**  **n=64** | ***P* value** |  | **Total**  **n=39** | **Discharged**  **n=24** | **No discharged**  **n=15** | ***P* value** |  |  |
| **MACE, n (%)** | 3(3.4) | 0(0) | 3(4.7) | 0.559 |  | 3(7.7) | 0(0) | 3(20) | 0.0498 |  | 0.370 |
| Index AMI | 1(1.1) | 0(0) | 1(1.6) | 1.000 |  | 0(0) | 0(0) | 0(0) | - |  | 1.000 |
| Subsequent AMI | 0(0) | 0(0) | 0(0) | - |  | 1(2.6) | 0(0) | 1(6.7) | 0.385 |  | 0.307 |
| Death | 0(0) | 0(0) | 0(0) | - |  | 0(0) | 0(0) | 0(0) | - |  | - |
| Emergency PCI | 0(0) | 0(0) | 0(0) | - |  | 1(2.6) | 0(0) | 1(6.7) | 0.385 |  | 0.307 |
| Urgent / elective PCI | 2(2.3) | 0(0) | 2(3.1) | 1.000 |  | 0(0) | 0(0) | 0(0) | - |  | 1.000 |
| CABG | 0(0) | 0(0) | 0(0) | - |  | 0(0) | 0(0) | 0(0) | - |  | - |
| Conservatively treated stenosis (>50%) | 0(0) | 0(0) | 0(0) | - |  | 2(5.1) | 0(0) | 2(13.3) | 0.142 |  | 0.093 |
| **Composite of death, AMI and emergency revascularization** | 1(1.1) | 0(0) | 1(1.6) | 1.000 |  | 1(2.6) | 0(0) | 1(6.7) | 0.385 |  | 0.522 |

AMI, acute myocardial infarction; CABG, coronary artery bypass grafting; HEART, History, ECG, Age, Risk factors, Troponin; MACE, major adverse cardiac events; PCI, percutaneous coronary intervention.

* HEART pathway (-) indicates combination of a HEART≤3 and negative serial cTn values (the first and second ones after presentation).

** *P* value for usual care (discharged) vs the potentially used HEART pathway (-).
